# Improvement of hypertension control and left-ventricular function after cure of primary hyperparathyroidism

**DOI:** 10.3389/fendo.2023.1163877

**Published:** 2023-07-10

**Authors:** Izabela Karwacka, Piotr Kmieć, Sonia Kaniuka-Jakubowska, Izabela Pisowodzka, Marcin Fijałkowski, Krzysztof Sworczak

**Affiliations:** ^1^ Department of Endocrinology and Internal Medicine, Medical University of Gdańsk, Gdańsk, Poland; ^2^ First Department of Cardiology, Medical University of Gdańsk, Gdańsk, Poland

**Keywords:** global longitudinal strain, left ventricular dysfunction, primary hyperparathyroidism (pHPT), hypertension, aldosterone, parathyroidectomy

## Abstract

**Introduction:**

Cardiovascular mortality is significantly higher in patients with primary hyperparathyroidism (PHPT) compared to the general population. The role of the renin-angiotensin-aldosterone system (RAAS) as a mediator of cardiovascular pathology in PHPT is unclear, as is the question whether successful parathyroidectomy (PTX) mitigates hypertension (HT), and left-ventricular (LV) dysfunction.

**Methods:**

In 45 consecutive, hypercalcemic PHPT patients (91% female, 20 normotensive, mean age 54.6 ± 14.6), laboratory examinations, and 24 h ambulatory blood pressure monitoring (ABPM) were performed before, one and six months after successful PTX, while transthoracic echocardiography (TTE) pre- and six months post-PTX.

**Results:**

Both in patients with normotension (NT) and HT, lower calcemia and parathyroid hormone (PTH) as well as higher phosphatemia were observed on follow-up, while B-type natriuretic peptide, aldosterone, plasma renin activity, and aldosterone-to-renin ratios were comparable. Six months post-PTX, only in patients with HT, median 24-hour SBP/DBP decreased by 12/6 mmHg, daytime SBP by 10, and nighttime DBP by 5 mmHg. Improvement in BP was observed in approximately 78% of patients with HT. Six months post-PTX, TTE revealed: 1) decrease in median LV mass index (by 2 g/m2) and end-diastolic dimension (by 3 mm) among patients with HT; 2) normalization of global longitudinal strain in 22% of patients (comparable between those with NT and HT); 3) a mean 12.7% reduction in left-atrium volume index among patients with HT, which underlay normalization of indeterminate diastolic function in 3 out of 6 patients with HT, who exhibited it at baseline (dysfunction persisted in 2).

**Conclusions:**

PTX was shown to significantly reduce BP, LV hypertrophy and diastolic dysfunction parameters in PHPT patients with HT, and improve systolic function in all PHPT patients.

## Introduction

Associations between primary hyperparathyroidism (PHPT) and cardiovascular (CV) diseases have not been studied extensively. Patients with PHPT are at an increased CV risk, and parathyroid hormone (PTH) has been postulated as an independent risk factor for CV diseases and sudden cardiac death (1, 2). CV diseases can be the main manifestation of both severe and mild or subclinical PHPT, yet, current endocrine guidelines do not account for hypertension (HT) or heart disease in the approach to management (3–6). Similarly, despite increased mortality due to CV diseases, PHPT is not discussed in cardiological guidelines.

HT is the most common CV complication of PHPT, and its prevalence is significantly higher in patients with this endocrinopathy than in the general population (7–9). Multiple mechanisms are involved in its pathogenesis, e.g. toxic effect of PTH on the vascular endothelium, dysfunction of vascular smooth muscles, vascular wall calcifications, and – possibly – stimulation of the renin-angiotensin-aldosterone system (RAAS) (8–16). Both elevated PTH and hypercalcemia affect the myocardium as well as vasculature. The former induces cardiac hypertrophy and positive chronotropic and inotropic effects (17–19). Hypercalcemia in the long-term leads to calcifications of the myocardium, valves and walls of vessels (18, 20). Elevated levels of PTH and calcium (Ca) are associated with higher arterial intima-media thickness (21–23). As a result, PTH increases the risk of myocardial ischemia and heart failure.

In the current study, we aimed at assessing the CV system in PHPT by investigating the effect of PTH and calcemia on blood pressure (BP), left-ventricular (LV) function as well as the RAAS in patients prior to and after successful parathyroidectomy (PTX).

## Subjects and methods

### Study participants and protocol

This study was approved by the local bioethics committee on July 2, 2015 (NKBBN/278/2015). Informed consent was obtained in writing from all study participants. Patients were recruited in the endocrine outpatient clinic of the Department of Endocrinology and Internal Medicine of the University Clinical Center of the Medical University of Gdańsk among those referred due to suspected or confirmed diagnosis of PHPT between 2015 and 2020.

The following inclusion criteria were adopted: age over 18, and diagnosis of PHPT based on the following laboratory criteria: serum PTH above 69 pg/mL (reference range 11–69) and total serum calcium (Ca) above 10 mg/dL (8.9–10). Exclusion criteria comprised: secondary and tertiary hyperparathyroidism, secondary HT, established or overt significant CV disease other than primary HT (in particular atherosclerotic CV disease including stroke, history of arterial revascularization, myocardial infarction, peripheral artery disease, as well as heart failure, clinically relevant valvular disease and arrhythmia), a lack of possibility of hypotensive therapy modification to medications non-interfering in the RAAS, active malignancy, poor physical condition, treatment with hypocalcemic drugs (e.g., biphosphates), albuminemia below 35 g/L, estimated glomerular filtration rate (eGFR) below 50 mL/min./1.73 m^2^, absence of indication or lack of patient’s consent for PTX.

In the period of the study, 45 patients were enrolled out of 85 referrals. PHPT was not confirmed in 21 patients (hyperparathyroidism was excluded in ten patients; eight and three were diagnosed with secondary and tertiary hyperparathyroidism, respectively), seven did not present for a follow-up visit, seven used biphosphates, three patients had contraindications to and two declined surgery.

The protocol consisted of: recording patient history and physical examination, additional examinations performed at baseline (prior to surgery), one and six months after successful surgical cure of PHPT. PHPT cure was defined as normal Ca along with normal or reduced by at least 50% PTH levels one month post-PTX. Laboratory tests and ambulatory blood pressure monitoring (ABPM) were performed before, ca. one and six months after successful surgical cure of PHPT, while transthoracic echocardiography (TTE) twice (before and six months after PTX). ABPM was conducted while patients were taking chronic hypotensive medications, after which a change to RAAS-non-interfering drugs (doxazosine and/or verapamil) for 14-18 days was made and laboratory tests were performed. In the case of examinations performed ca. one month post-PTX, ABPM was carried out between day 23 and 30, while laboratory assessment between day 37 and 45 after surgery. Directly (3-4 days) after ABPM, regular hypotensive drugs were re-introduced. Analogical assessment followed ca. 180 days post-PTX.

All patients had an enlarged parathyroid gland on sonography and/or scintigraphy. Histopathological diagnosis was mainly adenoma (63%), and less often hyperplasia. One female patient required two surgeries, since the first was unsuccessful.

### Laboratory examinations

Blood was drawn from an upper extremity vein between 8 and 10 a.m., centrifuged for 10 minutes at 12000 rpms, after which serum and plasma were extracted and kept at -20 degrees C. Dry ice was used to transport frozen samples to the Central Diagnostic Laboratory of the same hospital, where concentrations of PTH, Ca, phosphate (P), aldosterone (Ald), B-type natriuretic peptide (BNP) and plasma renin activity (PRA) were determined. A Siemens IMMULITE 1000 Immunoassay System was used to measure serum PTH concentrations; Ca, P and BNP concentrations were measured with an Abbott Architect analyzer (spectrophotometric method). Ald concentrations and PRA were determined by immunochemiluminescence using DiaSorin assays. Reference ranges recommended by manufacturers were adopted as normal. Ald-to-PRA ratio (ARR) below 30 ng/dL: ng/mL/h was considered normal.

### Office and ambulatory blood pressure measurements

The non-dominant arm was chosen for BP measurements. Office BP was measured using a validated device by Omron. Three measurements at one minute intervals were performed on every visit, and the mean of the second and third measurement was recorded. In-office BP was categorized in accordance with the ESC/ESH 2018 guideline as: optimal for measurements <120/80 mmHg, high normal for SBP 120-130 and/or DBP 80-84 mm, high normal for SBP of 130-139 and/or DBP of 85-89 mmHg, grade 1 for SBP 140-159 and/or DBP 90-99 mmHg, grade 2 for SBP 160-179 and/or DBP 100-109 mmHg, and grade 3 for SBP ≥180 and/or DBP ≥110 mmHg (24).

ABPM was conducted for at least 24 hours using a Spacelabs Ontrak 90227 monitor. Measurements were made every 15–20 minutes during daytime, and every 30 minutes at night. An arbitrary period between 10 p.m. and 6 a.m. was chosen for nighttime rest, and patients were obligated to follow it. If more than 30% of measurements were invalid, ABPM was repeated. ABPM results included mean 24-h, diurnal and nocturnal SBP and DBP as well as dipping status (normal provided mean nocturnal SBP and DBP were at least 10% lower than mean daytime readings). Normal SBP/DBP mean values acquired by ABPM were adopted from the 2018 ESC/ESH guideline: below 130/80 mmHg for the 24 h, below 135/85 mmHg for daytime, and below 120/70 mmHg for nighttime periods.

In follow-up assessment one and six months after surgery, improvement in BP was stated based on office and ambulatory measurements versus pre-PTX results if either of the following occurred: 1) cure of HT (office BP<140/90 mmHg, and normal mean SBP/DBP in ABPM without hypotensive medications), 2) better HT control, i.e., normalization of elevated pre-PTX 24-h, daytime and/or nighttime SBP/DBP with the same or lower number of hypotensive drugs, 3) comparable/lower 24-h, day- and/or nighttime SBP/DBP with fewer hypotensive drugs, and 4) dipping profile normalization.

### Echocardiography

Examinations were carried out on a GE Vivid E95 4D cardiovascular ultrasound machine in accordance with the Polish Society of Cardiology guidelines [24], [25]. Images were obtained typically in the parasternal long and short axis as well as apical, two-, three- and four-chamber projections. The following parameters were acquired: diastolic thickness of the interventricular septum (IVS), left ventricular end-diastolic dimension (LVEDd), left ventricular end-systolic dimension (LVESd), posterior wall thickness (PW), left-ventricular ejection fraction (LVEF), left atrial diameter (LAd), left atrial volume (LAV), left atrial volume index (LAVI), relative wall thickness (RWT), left ventricular mass (LVM), left ventricular mass index (LVMI), global longitudinal strain (GLS) and LV diastolic function parameters. RWT was calculated from the formula: RWT=2×PW/LVEDd.

We used the area-length method to calculate LVM. Measurements were performed at the end of diastole. Mean wall thickness was calculated from epicardial and endocardial cross-sectional areas in short-axis view at the papillary muscle level. LV mass was calculated from these measurements and LV length measured from the base to the apex. LVMI was indexed to body surface area (BSA) obtained from the Du Bois formula (BSA=0.007184 × W0.425 × H0.725, where W denotes body weight in kilograms, and H – height in meters): LVMI=LVM/BSA.

LAV was calculated by disk summation technique (modified biplane) from apical four and two chamber views. LAV was indexed to BSA (LAVI=LAV/BSA).

Four criteria for diastolic function assessment in patients with normal LVEF (>52% in men, >54% in women): 1) LAVI >34 mL/m^2^, 2) tricuspid regurgitation velocity (TR) >2.8 m/s, 3) early diastolic transmitral flow velocity (E)-to-averaged early diastolic mitral annular velocity (e’) ratio above 14 (E/e’>14), and 4) septal e’<7 cm/s or lateral e’<10 cm/s. In women with LVEF<55%, E was >50 cm/s and E-to-late diastolic peak velocity flow (A) ratio (or E/A) was below 2, therefore, three diastolic dysfunction criteria were assessed: E/e’>14, LAVI>34 mL/m^2^, TR>2.8 m/s [25].

GLS was measured by tracking acoustic markers in 3 standard projections (apical four-chamber, two-chamber, and long axis), and mean value was recorded. GLS was obtained from the formula: GLS (%)=100%×(MLs-MLd)/MLd, where MLs denotes myocardial length at late LV systole, and MLd – myocardial length at end-diastole. GLS above -19.5% (closer to zero) was considered abnormal.

### Statistical analysis

Statistical calculations were performed using GraphPad Prism software with one exception. Selection of statistical tools depended on the distribution of data. Kolmogorov-Smirnov test was used to test the normalcy of distribution. If normal, Student’s t-test was used for comparisons of two groups and repeated measures ANOVA with post-hoc Tukey’s test for paired data from three examination timepoints. To test data with non-normal distributions, U Mann-Whitney and Friedman’s (with *post hoc* Dunn’s multiple comparison) tests were used. Correlations were assessed using Pearson’s and Spearman’s methods depending on the distribution; their significance was verified with a dedicated test. Statistica version 14 software was applied to verify differences in binary variables at three examination timepoints by Cochran’s q test. Differences between two groups in binary variables were verified with Fisher’s exact test. Results are reported as number (%), arithmetic mean ± standard deviation (SD), and median (interquartile range, IQR). Significance was set at <0.05.

## Results

### Clinical data of study participants

Patients were predominantly female (41 versus 4), aged 54.6 ± 14.6 (range 25-80 years), with a mean BMI of 25.4 ± 5.2. Duration of PHPT based on anamnesis was 3.8 ± 2.8 years, its complications included nephrolithiasis in 29%, osteopenia in 18%, and osteoporosis in 33% of patients. Type 2 diabetes was present in 16 patients (36%), and hypercholesterolemia in 23 (51%); fifteen were active smokers.

Before enrollment, HT had been diagnosed in 22 patients (3.2 ± 1.8 years earlier – comparable to the time of PHPT diagnosis), while in 3 it was newly diagnosed based on office measurements and ABPM. A trend toward longer PHPT duration in patients with HT than NT could be observed (4.4 ± 2.7 versus 3.1 ± 1.7 years, p=0.06). Among the former, seven were aged below 50, eleven between 50 and 65, and seven 65 or older. Mean BMI of hypertensive patients was higher than that of normotensive: 26.9 ± 4.6 kg/m^2^ versus 23.5 ± 5.3, p=0.03, while mean age was similar (57 ± 13 vs 51 ± 16).

### Effect of parathyroidectomy on laboratory parameters

In one patient, calcemia equal to 10.9 mg/dL six months post-PTX resulted from iatrogenic calcium and alfa-calcidiol treatment and was fixed at 10 mg/dL. Two PRA values with corresponding ARRs were excluded from analysis due to a probable pre-laboratory error: 0.01 ng/mL/h pre-PTX in one participant, and 0.09 ng/mL/h one month post-PTX in another (both hypertensive females).

Prior to PTX, all patients met laboratory criteria for PHPT. After PTX, PTH and Ca decreased, while P increased significantly both in subjects with NT and HT (**Table 1**). Concerning RAAS parameters, there were three, three and four patients with an ARR>30, respectively, prior to, one and six months after surgery. However, in these patients, Ald and PRA values did not consistently indicate primary aldosteronism, i.e., at least at one point Ald was lower than 8 ng/dL and/or PRA exceeded 1 ng/mL/h. In contrast to calcium-phosphate parameters, initial BNP, Ald, PRA and ARR did not differ significantly from those on follow-up (**Table 1**).

**Table 1:** Laboratory parameters before and after PTX.

	All patients (n=45)				Patients with NT (n=20)				Patients with HT (n=25)			
	pre-PTX	1 and 6 months post-PTX		p	pre-PTX	1 and 6 months post-PTX		p	pre-PTX	1 and 6 months post-PTX		p
Ca [8.9-10 mg/dL]	11.5 ± 0.9	9.6 ± 0.6	9.5 ± 0.4	<10^-4^	11.9 ± 0.8	9.6 ± 0.6	9.4 ± 0.4	<10^-4^	11.3 ± 0.9^*^	9.6 ± 0.5	9.6 ± 0.4	<10^-4^
P [2.3-4.7 mg/dL]	2.2 ± 0.2	3 ± 0.6	3.1 ± 0.6	<10^-4^	2.2 ± 0.2	3 ± 0.4	3.1 ± 0.5	<10^-4^	2.2 ± 0.8	3 ± 0.7	3.2 ± 0.7	<10^-4^
PTH [10-69 pg/mL]	144 (91)	48.7 (41.1)	34.8 (27.1)	<10^-4^	192.2 ± 128.9	41.75 ± 22	36 ± 17.3	<10^-4^	140.6 ± 61.6	60.4 ± 42.2	42 ± 23	<10^-4^
BNP [<150 pg/mL]	16 (26.5)	17 (22.5)	18 (35.5)	n.s.	12.5 (15)	16.5 (17.3)	13 (14.8)	n.s.	27 (31.5)^#^	18 (24)	25 (44.3)^##^	n.s.
Ald [4.4-46.1 ng/dL]	10.5 (7.2)	10.5 (5.9)	9.5 (4.5)	n.s.	11.2 ± 5.5	10.6 ± 5.8	10.6 ± 5.4	n.s.	8.8 (8.1)	10.7 (7)	9.9 (6.2)	n.s.
PRA [ng/mL/h]	1.4 (1.6)	1.2 (1.7)	1.3 (2.2)	n.s.	1.4 (1.7)	1.2 (1.9)	1.3 (2.4)	n.s.	1.6 (5.1)	1.3 (3.2)	1.2 (3)	n.s.
ARR [<30]	6.5 (9)	8 (8.7)	7.2 (10.6)	n.s.	8.9 (6)	9.2 (7.7)	8.1 (6.8)	n.s.	4.1 (10.1)	6.9 (7.5)	6.3 (13.3)	n.s.

Data are presented as mean ± SD or median (interquartile range); p is given for repeated measures ANOVA or Friedman's test results; * pre-PTX, mean Ca was higher in patients with NT than HT (p=0.03, t Student test); # and ## pre- and 6 months post-PTX, median BNP was higher in patients with HT than NT (p=0.037 and 0.041, Mann-Whitney U test); n.s., not significant; PTX, parathyroidectomy.

Prior to PTX, there were statistically significant Pearson’s correlations between: PTH and Ca (R=0.54, p<0.0005), PTH and P (R=-0.42, p<0.005), Ca and P (R=-0.34, p=0.02). Neither before, nor after PTX were significant correlations found between Ca, PTH or P and BNP, Ald, PRA or ARR.

Pre-PTX, differences in Ald, PRA, ARR, and BNP were not found between patients in the lower half of Ca concentrations (<11.6 mg/dL) and others, nor between those with PTH lower than the median (144 pg/mL) and others.

P articipants with normotension (NT) had higher baseline calcium (11.9 ± 0.9 vs 11.3 ± 0.9 mg/dL, p=0.035), lower baseline and 6-month-post-PTX BNP, and a higher reduction in PTH one month post-PTX (150 ± 126 vs 80 ± 59 pg/mL, p=0.02) and Ca six month post-PTX (2.4 ± 0.9 vs 1.7 ± 0.9 mg/dL, p=0.01) than patients with HT (**Table 1**). These groups did not differ in Ald, PRA and ARR.

Among 20 patients with NT, no baseline correlations were found between Ca, P, PTH and Ald, PRA or ARR. There were also no statistically significant differences between pre- and post-PTX Ald, PRA and ARRs, nor a clear trend toward higher or lower levels. Similarly, in 25 patients with HT, no correlations were recorded between Ca, P or PTH and Ald, PRA or ARR, nor a trend hinting at possible differences (pre-surgery values were not significantly different from post-surgery ones, **Table 1**).

Finally, laboratory data were analyzed in hypertensive patients with and without BP improvement one month post-PTX (n=18 and 7, respectively). Baseline PTH was higher in those without improvement (180 ± 62 versus 125.3 ± 55.8 pg/mL, p=0.04). Among patients with HT and BP improvement, no trend was observed toward lower Ald or ARR, nor statistical significance upon testing for: a) differences in Ald, PRA and ARR at the three timepoints, b) correlations between Ald, PRA or ARR and Ca, P or PTH, nor c) correlations between pre- to post-surgery changes in RAAS and Ca-P parameters.

### Effect of parathyroidectomy on blood pressure

Overall, median daytime DBP was 5 mmHg lower one month post-PTX, and median 24-h SBP 9 mmHg lower six months post-PTX compared to baseline (**Table 2**). In patients with NT, ABPM results did not change post-PTX, whereas in patients with HT the following decreased significantly six months after surgery: 24-h SBP and DBP, daytime SBP, and nighttime DBP (**Table 2**). Mean change in 24-h, diurnal and nocturnal SBP as well as nighttime DBP from pre- to six-month-post-PTX values differed between patients with NT and HT (p between 0.01 and 0.04).

**Table 2:** Blood Pressure in 24-hour ambulatory monitoring before and after PTX.

	pre-PTX	1 month post-PTX	6 months post-PTX	p
All patients (n=45)				
24-h SBP	129 (21.5)	124 (19.5)	**120 (12)**	0.024^a^
24-h DBP	76 (17.5)	75 (12.5)	73 (12)	0.09
Daytime SBP	133 (22)	128 (20)	123 (14.5)	0.055
Daytime DBP	83 (19.5)	**78 (14)**	77 (12)	0.015^b^
Nighttime SBP	117 (29)	109 (19)	107 (13)	0.356
Nighttime DBP	67 (20)	67 (16)	65 (13)	0.156
Dipper status	27 (60%)	41 (91%)	41 (91%)	0.38
Patients with NT (n=20)				
24-h SBP	119.9 ± 13	118.2 ± 13.8	118.2 ± 10.9	0.54
24-h DBP	72.9 ± 10.4	71.35 ± 9.8	71.6 ± 8.9	0.92
Dipper status	12/16 (75%)	8/15 (53.3%)	11/15 (73.3%)	0.31
Patients with HT (n=25)				
24-h SBP	135 (33)	131 (17)	**123 (13)**	0.014^c^
24-h DBP	80 (24)	76 (12)	**74 (11.5)**	0.034^d^
Daytime SBP	138 (40)	135 (17)	**128 (13)**	0.042^e^
Daytime DBP	85 (27)	80 (10.5)	78 (11)	0.08
Nighttime SBP	129 (23)	118 (23)	109 (16)	0.31
Nighttime DBP	71 (28)	68 (12)	**66 (11.5)**	0.02^f^
Dipper status [n (%)]	16 (64%)	12 (48%)	16 (64%)	0.33

Data are presented as mean ± SD, median (IQR) or n(%); bold font indicates post-PTX values significantly different from pre-PTX; p for Friedman or repeated measures ANOVA test; superscript letters are used to indicate the following p in Dunn’s multiple post-hoc test: a and b – 0.034; c – 0.018; d – 0.049, e – 0.04, f – 0.022; HT – hypertension; NT – normotension; PTX – parathyroidectomy.

Regarding associations between BP and laboratory data, in all patients, the higher the decrease in PTH one month after PTX was, the lower 24-h, daytime SBP, DBP and nighttime DBP were (Pearson’s R between -0.43 and -0.35, p between 0.003 and 0.03). There were no correlations between ABPM and laboratory data in patients with NT and HT analyzed separately.

Among 25 patients with HT, there were 7 and 5 without improvement in BP, respectively one and six months after PTX, while in others the improvement consisted in: 1) cure of HT in 3 and 5 patients (1 with newly diagnosed HT), 2) better HT control (normalization of 24-h, day- and/or nighttime SBP/DBP) in 13 and 14 patients, 3) treatment with fewer hypotensive medications without an increase in BP in 7 and 9 patients, 4) change to dipper in 9 and 7 patients, respectively one month and six months post-PTX (**Table 3**). Taken together, improvement occurred in 18 patients (76%) one month, and 20 (80%) six months after surgery. Several patients met more than one criterion of BP improvement (e.g. 7 required fewer drugs to achieve better HT control).

**Table 3:** Change in blood Pressure in hypertensive patients after PTX.

Patients with HT (n=25)	1 month post-PTX	6 months post-PTX
Improvement in BP control	18 (72%)	20 (80%)
Cure of HT	3 (12%)	5 (20%)
Better HT control	13 (52%)	14 (56%)
Fewer drugs without higher BP	9 (36%)	6 (24%)
Change to dipper	3 (12%)	7 (28%)
No improvement in BP control	7 (28%)	5 (20%)
Change to non-dipper	7 (28%)	3 (12%)

Data are presented as n (%) and indicate change versus pre-PTX results, BP, blood pressure; HT, hypertension; PTX, parathyroidectomy.

Lastly, data of hypertensive patients with (n=18) and without (n=7) improvement in BP one month after PTX were analyzed. While pre-PTX, the former had higher 24-h SBP (146.2 ± 20.7 vs 125.7± 19.5 mmHg, p=0.03), nighttime SBP (134 ± 19.1 vs 114.3 ± 22.8 mmHg, p=0.04) and DBP (79.4 ± 13.8 vs 65.4± 18.4 mmHg, p<0.05), both one month and six months post-PTX 24-h, daytime SBP and nighttime SBP/DBP decreased by 10.8 to 22.6 mmHg in the former, while did not change significantly in the latter.

### Effect of parathyroidectomy on echocardiographic parameters

Considering all patients, LVEDd, LVM and LVMI decreased after PTX, which was attributable to those with HT (**Table 4**).

**Table 4:** Echocardiographic parameters before and six months after PTX.

	All patients (n=45)			Patients with NT (n=20)			Patients with HT (n=25)		
	pre-PTX	post-PTX	p	pre-PTX	post-PTX	p	pre-PTX	post-PTX	p
IVS [mm]	10 (3)	10 (3)	n.s	9.5 (2.8)	9 (2.5)	n.s	10 (2) ^*^	10 (2) ^**^	n.s
LVEDd [mm]	45 (6)	42 (3.5)	0.014	43.1 ± 5.1	42.8 ± 4.2	n.s	45 (7.5)	42 (7)	0.023
LVESd [mm]	29.2 ± 5.9	29.3 ± 5.3	n.s.	28.6 ± 3.9	29.4 ± 3	n.s	29.8 ± 7.2	29.3 ± 6.6	n.s
PW [mm]	9 (2)	9 (2)	n.s	9 (1)	8.5 (1)	n.s	10 (2) ^*^	10 (1) ^**^	n.s
LAd [mm]	35 (9)	34 (9)	n.s	32 ± 5.2	32.6 ± 4.6	n.s	38.2 ± 5.9 ^*^	37.6 ± 6.2 ^**^	n.s
LAV [ml]	49.1 ± 19.2	46.1 ± 14.9	n.s	37.7 ± 10.2	39.8 ± 11.3	n.s	58.3 ± 19.9^*^	51.1 ± 15.6^**^	0.036
LAVI [ml/m²]	27.9 ± 11.5	25.8 ± 7.8	n.s.	21.8 ± 5.9	22.4 ± 5.9	n.s.	32.8 ± 12.6^*^	28.6 ± 8 ^**^	0.043
Normal diast. function [n(%)]	37(82.2%)	40(88.9%)	n.s	20 (100%)	20 (100%)	n.s.	17(68%) ^*^	20 (80%)	n.s.
LVEF [%]	58 (6)	59 (4)	n.s	58 ± 3.2	58.2 ± 2.7	n.s	59 ± 7.1	59.7 ± 6.9	n.s
LVM [g]	109 (50)	103 (40)	<10^-3^	103 (33)	101 (50)	n.s	117 (49)	104 (31)	10^-3^
LVMI [g/m²]	63.3(19.5)	61.2 (14.1)	10^-3^	63.5 ± 11.6	63.6 ± 12.9	n.s	63.6 (22.4)	60.2 (11.8)	10^-3^
E/e`	9.13 ± 2.76	9.1 ± 2.44	n.s	8.78 ± 2.5	8.6 ± 2.3	n.s.	9.41 ± 2.97	9.5 ± 2.52	n.s.
RWT [mm]	0.44 ± 0.09	0.45 ± 0.1	n.s	0.42 ± 0.1	0.41 ± 0.08	n.s	0.46 ± 0.08	0.48 ± 0.1	n.s
GLS [%]	-19 (3.5)	-20 (2)	<0.01	-18.5 (2)	-20 (2)	<0.01	-19 (4)	-20 (2.5)	n.s
Normal GLS [n(%)]	17 (38%)	27 (60%)	10^-3^	8 (40%)	13 (65%)	0.02	9 (36%)	14 (56%)	0.02

mean ± SD, median(IQR) or n(%) are presented; ^*^ and ^**^ denote statistically significant differences between patients with NT and HT, respectively pre- and post-PTX, p between 0.0004 and 0.007; diast., diastolic; GLS, global longitudinal strain; GLS was considered normal when equal to or lower (more negative) than -19.5%; HT, hypertension; n.s., not significant; NT, normotension; PTX, parathyroidectomy.

There was no statistically significant change in LVEF in patients with NT nor with HT. Reduced LVEF was recorded in four women pre-PTX (45%, 45%, 50%, and 52%), and remained so in two post-PTX (48%, 50%, 57%, and 58%, respectively).

Before surgery, normal (below -19.5%) GLS was recorded in 17 patients (37.8%), which improved to 60% six months post-PTX. In contrast to hypertrophy parameters, GLS was lower (better) in patients with NT after surgery, but not in those with HT. Still, also in the latter normal GLS following PTX was recorded more frequently (**Table 4**).

Concerning diastolic function, it was normal in patients with NT at both timepoints, while a 12.7% reduction in LAVI was observed post-PTX in patients with HT. A trend toward fewer instances of indeterminate diastolic function after surgery (6 versus 3) could be observed in the latter, while diastolic dysfunction persisted in 2 (**Table 4**).

No significant correlations were found between laboratory and echocardiographic parameters. Ald and ARR decreased and increased in a similar number of patients, therefore, correlations with TTE parameters were not tested.

In subjects with HT, baseline PTH correlated with IVS (Spearman’s r=0.6, p=0.002) and PW (r=0.48, p=0.016). Upon analyzing 18 hypertensive patients with improvement in BP one month post-PTX, no statistically significant correlations between changes in laboratory and echocardiographic parameters were found; however, LVM and LVMI correlated with changes in 24-h and daytime SBP and DBP (Spearman’s r between 0.44 and 0.57, p between 0.014 and 0.046).

## Discussion

Our data indicate that surgical cure of PHPT reduces BP and LV hypertrophy among hypertensive patients, as well as improves cardiac function both in normo-, and – more markedly – in hypertensive patients, independently of the RAAS. BNP remained higher in patients with HT than with NT, which is probably attributable to higher LAVI in the former. Strong points of our study include a clearly defined patient population, careful preparation, and thorough evaluation of patients (clinical, laboratory, ABPM, and TTE assessment). Limitations include lack of controls, small sample size considering wide range of age, patient heterogeneity as well as a rather short follow-up period.

Thus far, discordant findings have been reported concerning the reversibility of HT or BP reduction following the cure of PHPT. Ejlsmark-Svensson et al. compared PHPT patients randomized to observation (n=36) or surgery (n=33), and found BP did not change three months after PTX in the latter (25). In a larger, randomized controlled trial, two years post-PTX, a decrease in DBP and mean BP was observed in operated (n=54) but not observed (n=62) patients, however, with no significant difference between these groups (26). Unchanged BP values were also recorded after surgery in two small observational studies (n=48 and 30, respectively) by Karakose et al. and Nilsson et al. (14, 27). In these four studies, patients with NT and HT were not analyzed separately, which is vital, since the effect of PTX on BP was apparent only in the latter in several other reports. This is indicated by findings from a large retrospective cohort (n=1020) and a smaller one (n=368): six months post-PTX, SBP and DBP decreased in patients with HT (71% and 50%, respectively) but not those with NT (28, 29). Our data also point at the beneficial effect of surgery on BP in hypertensive patients only. However, there are reports with contrasting results. For instance, Rydberg et al. observed 5 mmHg higher SBP in 20 PHPT patients with HT after PTX, yet, they were older, PHPT and HT had lasted longer, and hypercalcemia was milder than in our patients. In turn, Beysel et al. reported a BP reduction in both 35 normo- and 60 hypercalcemic patients (43% and 63% with HT, respectively) six months after successful surgery (13, 30).

Similarly to data concerning BP, improvement in echocardiographic parameters after PTX recorded in our study (LVH reduction, better systolic and diastolic function) can only be partially tracked to previous studies. In particular, at the same six-month follow-up: Agarwal et al. found LVEF and diastolic function (E/A ratio) improved, Kepez et al. reported better LV contractility by speckle tracking examination but no change in LVM or diastolic function, while in patients randomized to surgery (n=12) or observation (n=12) by Pepe et al. neither systolic nor diastolic cardiac function altered (31–33). Two meta-analyses (from 2015 and 2017) contain discordant findings: a decrease in LVM was reported by McMahon et al. in short-term follow-up (up to 6 months) based on 15 prospective studies (11 observational, 4 randomized, n=457), while Best and colleagues found no differences in LVEF or IVS, RWT, and two diastolic function parameters upon analyzing 14 studies (3 randomized, n=424) (34, 35). Heterogeneity of the designs and patient populations (in particular concerning CV risk factors and PHPT severity) probably account for divergent results. It is worth noting that in our study a decrease in LVM and LAVI was recorded in hypertensive, while GLS improved more markedly in normotensive patients.

PTH excess present in PHPT has been implicated as a stimulatory factor for the RAAS. In particular, several studies documented lower Ald, PRA and/or angiotensin II after PTX (36). The current study does not confirm this: neither before nor after PTX were there significant abnormalities in the RAAS parameters, and no change was recorded in Ald, PRA and ARR in patients with NT nor HT following surgery. Similar findings were reported by Bernini et al., and Salahudeen et al., as well as Castellano et al. in normotensive PHPT patients (37–39). Multiple factors affecting the RAAS significantly complicate investigations of possible relationships with calcium-phosphate parameters, e.g. patient populations, medications (especially hypotensive drugs), age, presence of HT, hydration, laboratory assays, etc. Mild primary aldosteronism concomitant with PHPT should also be taken into consideration in light of partly similar demographic (age above 50) (40).

## Conclusions

PHPT is nowadays rarely diagnosed in patients with classic symptoms, hence, data on HT and impaired cardiac function due to this endocrinopathy gain importance. In our study, normotensive patients with PHPT had comparable ABPM results but better systolic function post-PTX, while those with HT exhibited lower BP and LV hypertrophy parameters, improved systolic and diastolic function on follow-up. These significant cardiovascular benefits of surgical cure of PHPT require further confirmation in larger studies.

## Data availability statement

The raw data supporting the conclusions of this article will be made available by the authors, without undue reservation.

## Ethics statement

The studies involving human participants were reviewed and approved by Independent Bioethics Committee for Research of the Medical University of Gdańsk. The patients/participants provided their written informed consent to participate in this study.

## Author contributions

IK, KS and SK-J contributed to conception and design of the study. IK, SK-J, KS, IP and MF contributed to data acquisition. IK and PK organized the database. PK performed the statistical analysis. IK and PK wrote the first draft of the manuscript. All authors contributed to manuscript revision, read, and approved the submitted version.
